# Wnt-β Catenin Signaling Pathway: A Major Player in the Injury Induced Fibrosis and Dysfunction of the External Anal Sphincter

**DOI:** 10.1038/s41598-017-01131-6

**Published:** 2017-04-19

**Authors:** M. Raj Rajasekaran, Sadhana Kanoo, Johnny Fu, Valmik Bhargava, Ravinder K. Mittal

**Affiliations:** Department of Medicine, Division of Gastroenterology, San Diego VA Health Care System & University of California, San Diego, CA USA

## Abstract

Wnt-β catenin is an important signaling pathway in the genesis of fibrosis in many organ systems. Our goal was to examine the role of Wnt pathway in the external anal sphincter (EAS) injury-related fibrosis and muscle dysfunction. New Zealand White female rabbits were subjected to surgical EAS myotomy and administered local injections of either a Wnt antagonist (sFRP-2; daily for 7 days) or saline. Anal canal pressure and EAS length-tension (L-T) were measured for 15 weeks after which the animals were sacrificed. Anal canal was harvested and processed for histochemical studies (Masson trichrome stain), molecular markers of fibrosis (collagen and transforming growth factor-β) and immunostaining for β catenin. Surgical myotomy of the EAS resulted in significant impairment in anal canal pressure and EAS muscle L-T function. Following myotomy, the EAS muscle was replaced with fibrous tissue. Immunostaining revealed β catenin activation and molecular studies revealed 1.5–2 fold increase in the levels of markers of fibrosis. Local injection of sFRP-2 attenuated the β catenin activation and fibrosis. EAS muscle content and function was significantly improved following sFRP-2 treatment. Our studies suggest that upregulation of Wnt signaling is an important molecular mechanism of injury related EAS muscle fibrosis and sphincter dysfunction.

## Introduction

Fecal incontinence (FI) is defined as the inability to control bowel movements causing accidental, unintentional loss of solid or liquid stool from the rectum. It is extremely common, known to have devastating effect on the quality of patient’s life and is a common cause for institutionalization in the elderly. The negative impact of FI on the woman’s physical and emotional health is tremendous including reluctance to consider future pregnancies^1, 2^.

While the pathophysiology of FI is multifactorial; injury to the anal sphincters, surgical and childbirth related, is an important cause. The vaginal childbirth related injuries to the anal sphincter and pelvic floor muscles are extremely common. New onset of FI symptoms have been reported in up to 44% of women after vaginal delivery. Nine percent of women suffer from a 3^rd^ or 4^th^ degree tears during vaginal delivery and 2/3^rd^ of them suffer from FI subsequently. Occult injuries to the anal sphincter (OASIS) and puborectalis muscle have been found in 25–35% women after vaginal delivery. Less well understood is the relationship between occult injury to the anal sphincter muscle and late onset of FI symptoms, i.e., 2–3 decades later^3–6^. The prevalence of FI appears to be same in men and women but the severe form of FI occurs predominantly in women as suggested by the observations that the majority of participants in clinical trials of FI, medical and surgical, are actually women, which we suspect is related to obstetrical injuries. Manometric data show poor voluntary squeeze pressures of the anal canal in 73% of patients with FI symptoms^7^. We found that the length-tension function of the EAS is impaired in majority of women with FI^8^. Therefore, we suspect that injury to the EAS muscle is a major risk factor in the pathogenesis of FI.

We found that following surgical myotomy of the EAS muscle in rabbit, there is marked impairment of its function (length-tension function) and the anal canal pressure^9^. Histological evaluation revealed that the spontaneous repair following surgical myotomy to the EAS muscle occurs not by replacement with normal muscle, instead with fibrous tissue^9^. The molecular mechanism(s) that dictate whether an injury heals by fibrosis or muscle is not known. Wnt-β catenin signaling pathway is a major player in the injury related fibrosis in several organ system, i.e., myocardium, lungs, kidney, liver and skin^10^. The Wnt/β-catenin signaling is also known to regulate the expression of an important fibrogenic growth factor (TGF-β) and TGF-β1 can promote β-catenin signaling^11–13^. The latter has also been implicated in the age related fibrosis and dysfunction in the skeletal muscle^14^.

The goal of our study was to evaluate the role of Wnt-β catenin signaling pathway in the surgical myotomy related fibrosis and dysfunction in the EAS muscle. We achieved the above by determining myotomy related alterations in the structure and function of the EAS muscle including levels of fibrogenic markers (β-catenin, Collagen-I and TGF-β1). We also examined the effects of a specific Wnt antagonist by local injection of sFRP-2 (secreted frizzled related protein-2) on the EAS muscle structure and function, following a surgical myotomy in the rabbit model.

## Results

### Effect of EAS surgical myotomy and Wnt antagonist (sFRP-2) on the anal canal function

**Figure 1:**
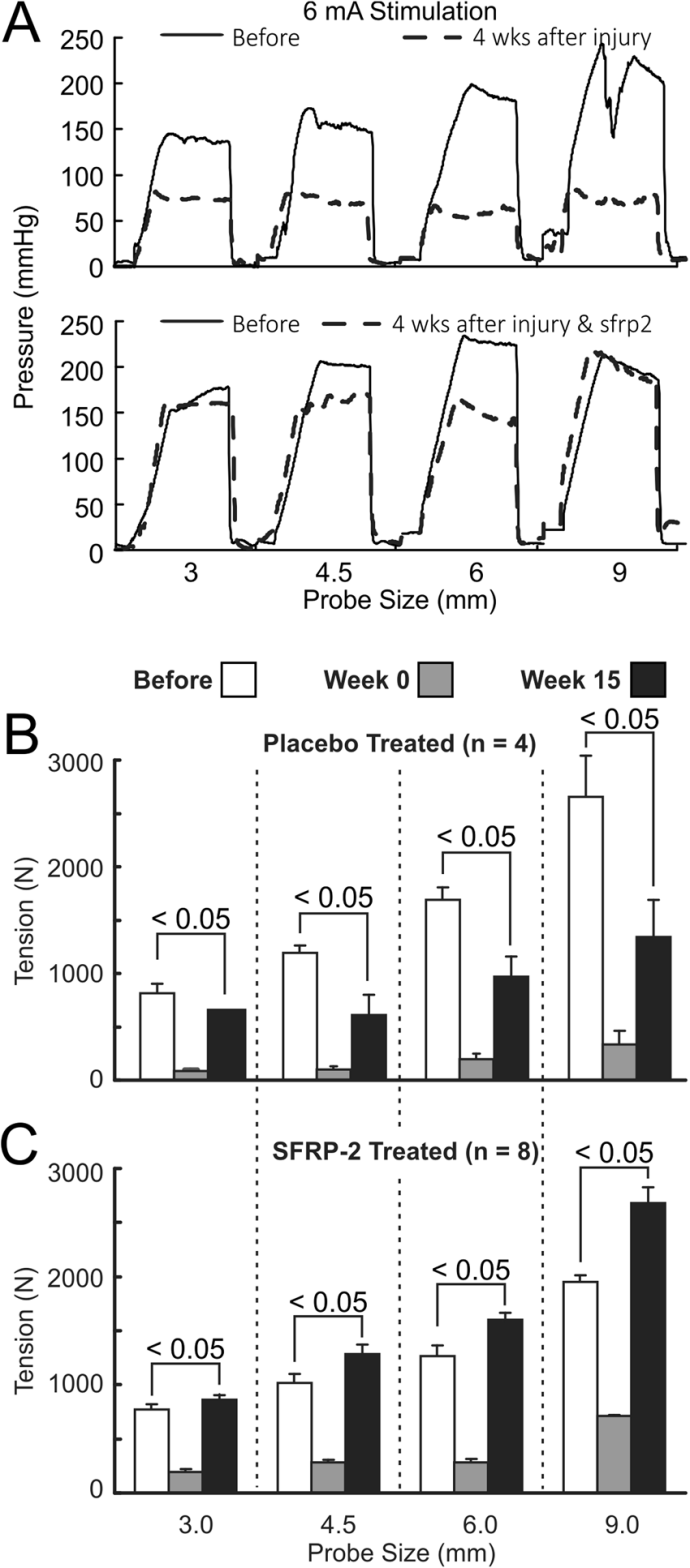
(**A**) Representative rabbit anal canal pressure tracings before and after EAS myotomy measured with probe sizes of 4.5, 6 and 9 mm diameter from animals treated with placebo (top panel) or Wnt antagonist (bottom panel) after 21 days post-treatment; (**B**) Effect of EAS myotomy on its length-tension relationship at 0 and 15 weeks post-myotomy. Muscle tension values from animals treated with placebo are shown. A significant decrease (P < 0.05) in EAS tension when compared to pre-injury values were observed for all probes in untreated animals. Panel (C) shows values from animals treated with a Wnt antagonist (sFRP-2; 2 µg × 7 days). A significant improvement (p < 0.05) in EAS tension for probes 4.5, 6 and 9 mm was observed in sFRP-2 treated animals at 15 weeks post-myotomy compared to pre-myotomy values.

Figure 1A shows representative tracings of the effects of EAS myotomy and electrical stimulation on the anal canal pressure. Top panel shows the anal canal pressure measured with different size probes before (solid black line) and after myotomy (dotted black line) in the placebo treated (control) animal. The bottom panel shows tracings from a sFRP-2 treated animal 4 weeks post-injury. After myotomy, the anal canal pressure does not increase with the increase in probe size in the placebo treated animal. On the other hand, in the sFRP-2 treated animals the anal canal pressure and length-tension function returns to the near normal pre-myotomy values. Figure 1B,C shows the length-tension property of the EAS muscle before, immediately after, and 15 weeks post-myotomy in the placebo and sFRP-2 treated animals. Prior to myotomy, maximal electrical stimulation of the EAS produced a steep, probe-dependent increase in the muscle tension (open bars), which was not the case immediately after myotomy (grey bars). At 15 weeks post-injury, a significant decrease in the EAS muscle tension was observed with all four probe sizes as compared to before myotomy. The muscle tension (mN/cm^2^) before myotomy in the placebo treated animals for 3, 4.5, 6 and 9 mm probes were 787 ± 99, 1123 ± 12, 1658 ± 118 and 2688 ± 17 mN/ cm^2^, respectively. Immediately after myotomy, the tension dropped and remained low compared to before myotomy at the end of 15 weeks (3, 4.5, 6, and 9 mm probes) in the placebo treated animals. In the treatment group, the values before myotomy for the 3, 4.5, 6, and 9 mm probes were 771 ± 50, 1018 ± 80, 1265 ± 1, 1950 ± 60 mN/cm^2^ and at the end of 15 weeks (post-myotomy and treatment with sFRP-2) the muscle tension values improved and were comparable to the pre-myotomy values, (857 ± 50, 1283 ± 80, 1599 ± 6, 2674 ± 10 mN/cm^2^ for the 3, 4.5, 6, and 9 mm probes respectively).

**Figure 2:**
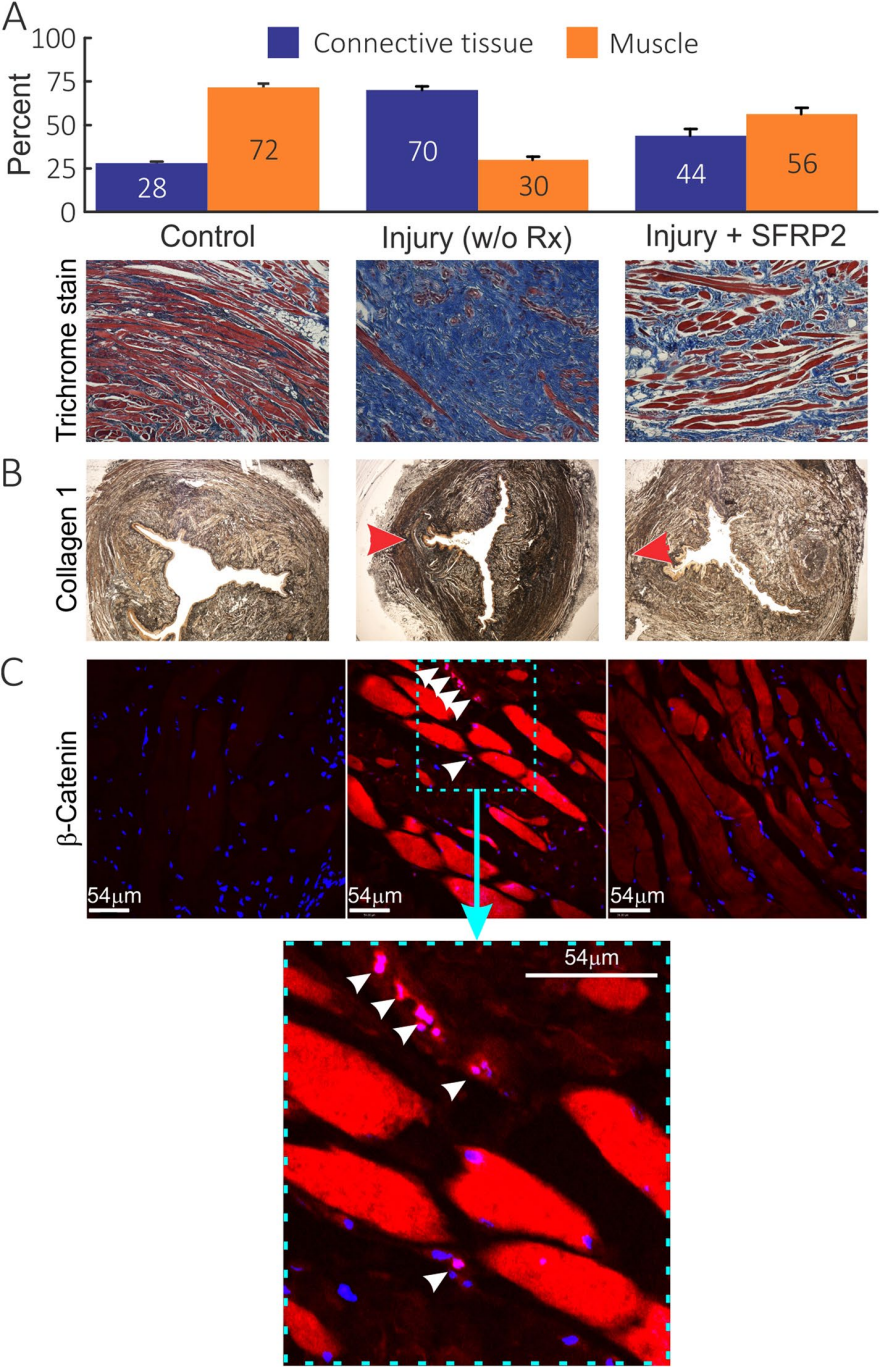
(**A**) Masson- trichrome stained rabbit EAS tissues from uninjured control (left), injured and treated with placebo (middle), injured and treated with sFRP-2 at 15 weeks post-myotomy. Histological evidence of sustained and widespread fibrosis is demonstrated with Masson- trichrome stain in placebo treated animals. The EAS muscle is stained in red and connective tissue in blue in these images. A significant increase in connective tissue and a decrease in muscle was observed in untreated animals (P < 0.05) compared to uninjured controls (n = 3–4 from each group). (**B**) Photomicrographs of rabbit anal canal cross sections showing collagen-I protein expression from control (left), injured and treated with placebo(middle), injured and treated with sFRP-2 at 15 weeks post-myotomy. Increased collagen (intense brown) is seen in tissues from animals treated with placebo and this increase was attenuated in sFRP-2 treated rabbits. Red arrows indicate site of surgical myotomy. (**C**) Photomicrographs of rabbit anal canal cross sections showing β-catenin protein expression from control (left), injured and treated with placebo (middle), injured and treated with sFRP-2 at 15 weeks post-myotomy. Increase in β-catenin nuclear activation (white arrow heads) is seen in tissues from animals treated with placebo and this increase was attenuated in sFRP-2 treated rabbits. Inset shows nuclear activation.

Figure 2A shows representative photomicrographs of the anal canal cross sections stained with trichrome from controls (no myotomy but sham surgery), placebo and sFRP-2 treated animals. In these sections, red color represents muscle and blue is the connective tissue. In controls animals, the anal canal cross section shows EAS muscle fibers (72%) arranged in a uniform pattern with relatively small amount of connective tissue (28% connective tissue) (Fig. 2A). In animals subjected to myotomy and treated with placebo, there was significant decrease (P < 0.05) in the muscle content (30% muscle) and increase in the fibrosis (70% connective tissue). On the other hand, in the animals treated with sFRP-2, there was significant decrease in the fibrosis (44% connective tissue) and improvement in muscle regeneration (56% muscle content) at 15 weeks. In addition, staining with collagen-I specific antibody confirmed increase in the collagen-I levels (Fig. 2B; an intense brown stain in the middle-panel, red arrow head) after EAS myotomy that was attenuated by sFRP-2 treatment (Fig. 2B; right panel). The middle panel (myotomy w/o Rx) shows an increase in collagen-1 expression after myotomy which was attenuated by SFRP 2 treatments (right panel). We also performed immunofluorescence studies (to confirm β-catenin nuclear activation) and immuno-histochemical analysis to confirm collagen- I changes in the harvested specimens to investigate the effect of myotomy and efficacy of sFRP-2. Specifically, the EAS myotomy increased β-catenin nuclear activation (Fig. 2C- middle panel, white arrow heads). On the other hand, treatment with the sFPR2 attenuated myotomy induced β-catenin nuclear activation (Fig. 2C- right panel).

**Figure 3:**
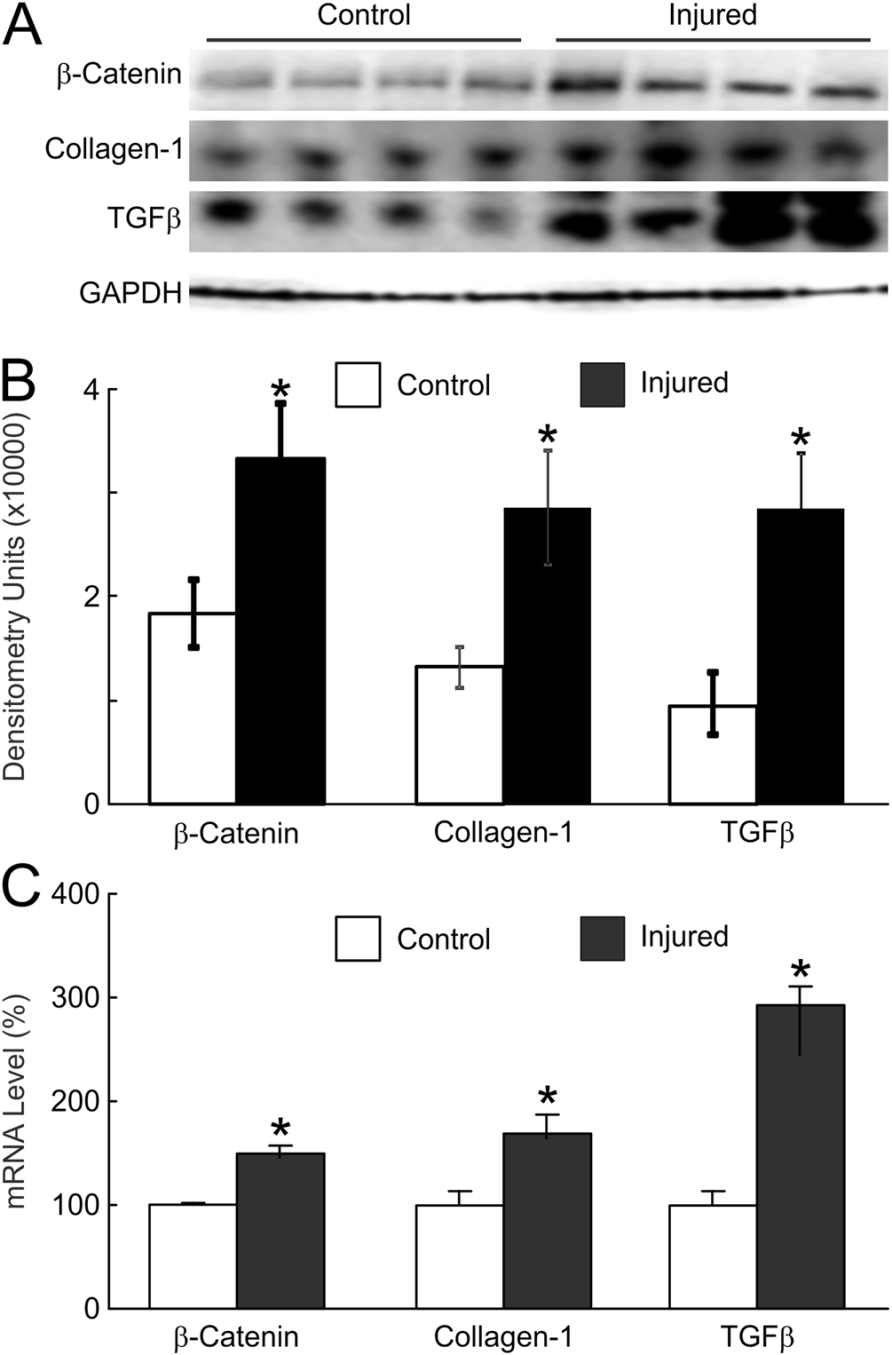
Rabbit EAS tissue protein levels estimated by Western blot (**A**) followed by (**B**) densitometry and (**C**) mRNA levels (bottom) of β-catenin collagen-I, and TGF-β from control and animals subjected to EAS myotomy estimated at 15 weeks post-myotomy (3–4 from each group). Full-length blots/gels are presented in Supplementary Figure 1.

Figure 3 (panels A,B) show representative images depicting injury related changes in the protein levels of important fibrogenic proteins, β- catenin, collagen-I and TGF-β. After EAS myotomy there was significant increase in all three fibrogenic proteins (Fig. 3B). The injury related changes in the mRNA levels of, β-catenin, collagen-I and TGF-β in the EAS injured samples was compared to the non-injured control animals. We found significant increase (1.5–3-fold) in the expression of fibrosis markers in the myotomy group compared to controls (Fig. 3C **)**.

## Discussion

The results of this study confirm our previous findings that the EAS surgical myotomy results in, (1) loss of muscle function, i.e., decrease in anal canal pressure and alteration in EAS length-tension property, and (2) spontaneous EAS repair following EAS myotomy occurs through excessive collagen deposition and reduction in muscle mass^9^. The new findings of our studies are, (1) following EAS myotomy, there is upregulation of Wnt-β catenin and TGF-β signaling pathways, (2) we monitored the sphincter muscle healing after interventions with a Wnt antagonist (sFRP-2) for 15 weeks post-myotomy. We evaluated EAS muscle function by determining its length-tension property, fibrosis and collagen deposit in the muscle and changes in fibrogenic markers. Intervention with an antagonist (sFRP-2) suppressed fibrosis and improved muscle function. Overall, our results reveal involvement of Wnt-β catenin signaling pathways in mediating surgical myotomy induced fibrosis and EAS muscle dysfunction.

After injury, the skeletal muscles such as EAS regenerate through a series of well-coordinated events^15^. The regeneration starts with activation of quiescent satellite cells that reside in the muscle. The later leads to formation of new muscle fibers, which go through proliferation and differentiation^16^. Major events of the healing processes are: (1) an initial inflammatory reaction & invasion of macrophages, (2) activation, differentiation & fusion of satellite cells & (3) maturation of newly formed myofibers and remodeling of regenerated muscle. There are several important signaling pathways that regulate muscle repair and among them the Notch and canonical Wnt signaling^17–19^ are thought to be critical. Notch pathway is believed to be involved in the myoblast proliferation and Wnt signaling modulates differentiation process. Canonical Wnt signaling cascade involves soluble Wnt ligands interaction with Frizzled receptors and low-density lipoprotein receptor-related protein co-receptors (LRP). The later stimulates phosphorylation of disheveled (a cytosolic phosphoprotein) protein that inactivates glycogen synthase kinase 3 β (GS3Kβ) phosphorylation of β catenin. With the assistance of axin, the de-phosphorylated and stable β- catenin does not undergo ubiquitination and degradation; instead it is translocated to the nucleus where it binds to TCF/LEF1 transcription factors^20–22^.

Wnt/β-catenin signaling is activated as a consequence of injury and is reported to play a major role in the several types of injuries induced fibrosis in several organ system, i.e., ischemia induced myocardial fibrosis, idiopathic and bleomycin induced pulmonary fibrosis, fibrosis seen in chronic kidney failure, liver fibrosis and abnormal skin wound healing^12, 23^. In all of the above examples Wnts and positive regulators of β-catenin signaling are up-regulated and inhibitors of Wnt/β-catenin signaling are down-regulated. Antagonists of Wnt have been found to reduce fibrosis in the above organ systems. We used the secreted frizzled receptor protein-2 (sFRP-2), a Wnt antagonist to intervene in the EAS muscle myotomy induced anal sphincter dysfunction and muscle fibrosis. Our finding is that similar to other organ systems, the Wnt signaling pathway also plays a major role in the EAS muscle repair/regeneration. Our findings are in agreement with the findings of others that skeletal muscle injury up-regulates the Wnt signaling pathway^14, 24, 25^. The dose and route of administration of sFRP-2 in our study was based on a recently published report in the rat model^26^ that studied myocardium. The time points that we tested were are also based on the literature which showed improvement by 30 days post-treatment^26^. The sFRPs structurally resemble Wnt frizzled receptor and can inhibit β-catenin signaling by working as Wnt-decoy receptor^27^. The sFRP-2 may act by a dual mechanism of fibrogenesis, (1) antagonize Wnt receptor engagement, and (2) inhibit collagen processing/maturation. Akhmetshina *et al*.^11^ found that canonical Wnt signaling is critical for the TGF-β-mediated fibrosis and implicate a key role for the interaction of both pathways in the pathogenesis of fibrotic diseases. Findings from our study that the EAS myotomy related fibrosis involves both Wnt and TGF-β-mediated signaling pathways support these observations. Targeted anti-fibrotic therapy has been shown to reduce fibrosis and improve organ function.

Injury to the EAS muscle in humans may result from several possible etiologies, (1) surgical trauma, i.e., following operations in this area for anal fissure and hemorrhoids, (2) accidental and sports injury (e.g. goring during bull fight) and (3) most commonly from obstetrical trauma. The latter is extremely common and can be related to excessive stretch during spontaneous passage of fetus through the birth canal, use of forceps and surgical episiotomy. The disruption of anal sphincter muscles at the time of vaginal delivery may be obvious or occult (OASIS). One can argue that our model of muscle injury, i.e., surgical EAS myotomy may not be completely representative of what happens during various types of injuries. However, our study is the first one to show the role of Wnt signaling in the EAS muscle injury and repair, and should stimulate other studies to investigate whether similar mechanism is operative in other types of injuries to the anal sphincter and pelvic floor muscle. If proven to be correct, our findings have tremendous clinical implications, i.e., Wnt antagonist may be useful in preventing EAS and other pelvic floor muscle dysfunction following surgical and obstetrical injuries, which are extremely common and have devastating consequences. A number of Wnt antagonists are available for systemic, oral and topical use^10^, and merit clinical trials to prevent trauma (surgical, sports and obstetrical) induced anal sphincter muscle dysfunction. We propose that the Wnt antagonists are novel potential therapeutic agents for the prevention of anal sphincter dysfunction resulting from various types of anal sphincter injuries.

Our study, however, has a few limitations: (1) we did not measure the protein and mRNA levels of β-catenin, collagen-I and TGF-β after sFPR2 treatment that would have provided additional evidence for the mechanism of action of frizzled receptor antagonist in our model of injury induced fibrosis. However, based on several current reports, it is clear that the sFRPs act as a Wnt-decoy receptor due to their structural resemblance to Wnt frizzled receptor to inhibit β-catenin signaling^26–29^. (2) Our data only suggests but does not entirely prove that the Wnt-β catenin is necessary for the EAS injury-related fibrosis, which would require additional *in-vivo* and *in vitro* experiments and that is the goal of our future studies. (3) We only tested one Wnt antagonist in our study; administration of other antagonists to inhibit downstream targets of Wnt^10, 30, 31^ may be helpful to elucidate if and how other agents could reduce fibrosis and improve EAS function.

## Materials and Methods

The institutional animal care and use committee at the VA San Diego Healthcare Systems approved the study protocol and all experiments were conducted in accordance with the Guidelines and Use of Laboratory Animals (National Institutes of Health, Bethesda, MD). Adult New Zealand white female rabbits (n = 18; 4–5 kg) were anesthetized with an intramuscular injection of ketamine (35 mg/kg) and xylazine (5 mg/kg). Of these, fourteen rabbits were subjected to EAS myotomy and four were subjected to sham surgery (uninjured controls). These control animals were maintained and sacrificed at the end of the study (15 weeks) and EAS muscle tissue was used for histological and molecular analysis.

**Figure 4:**
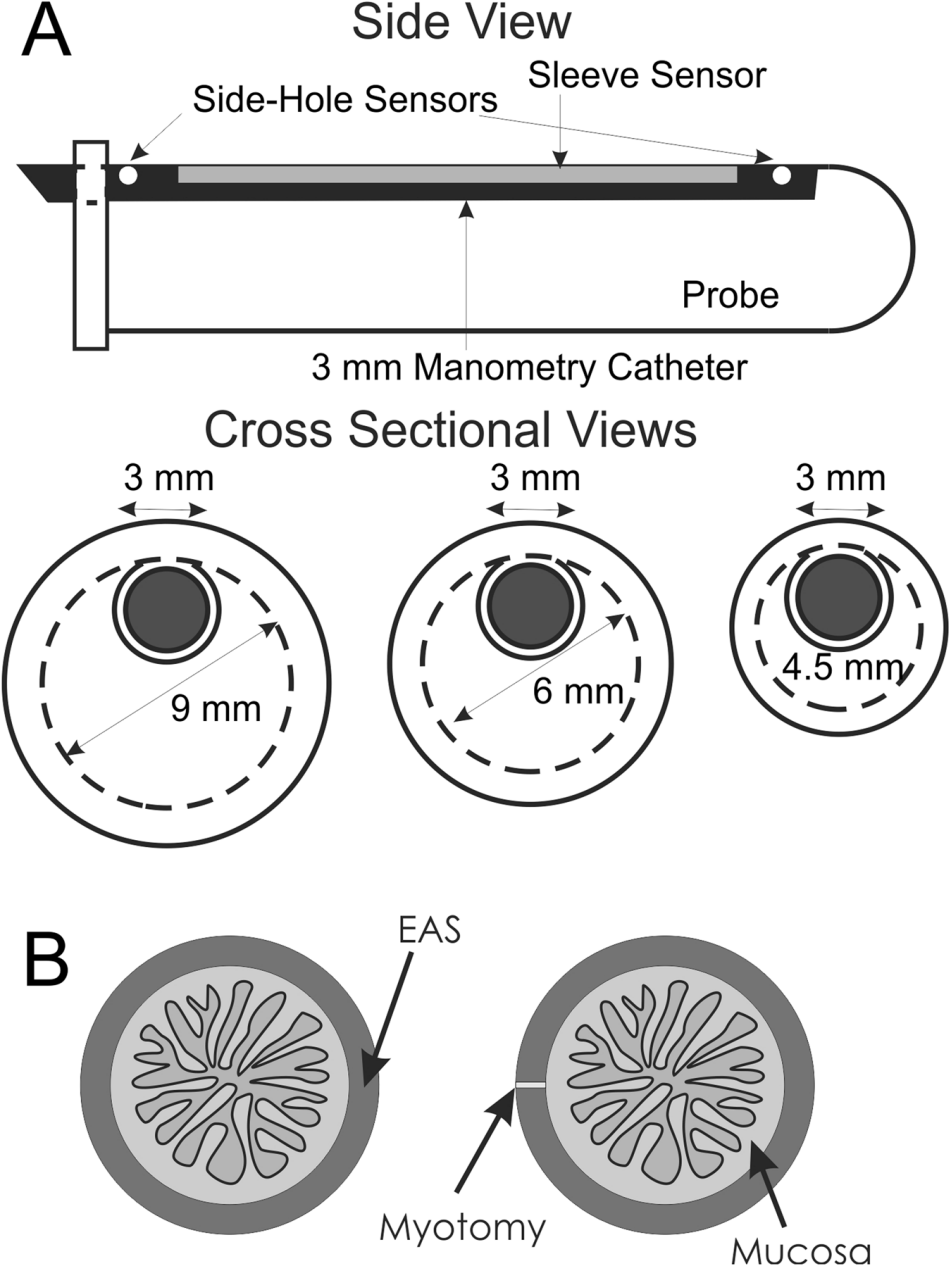
Schematics of (**A**) custom designed probe holders of 4.5, 6 and 9 mm diameter used in the EAS length-tension studies, and (**B**) schematic of rabbit external anal sphincter myotomy.

Two custom designed copper wire hook electrodes were placed in the EAS muscle for electrical stimulation. Anal canal pressure was measured using manometric methods; a 3 mm diameter sleeve sensor was placed in custom designed probe holders of 4.5 mm, 6 mm and 9 mm diameter, Fig. 4A. EAS muscle was subjected to electrical field stimulation and anal canal pressure was recorded as described previously^32, 33^. Ultrasound images of anal canal were obtained to determine the anal canal radius and EAS muscle thickness. Ultrasound imaging of anal canal was performed obtained to determine the anal canal radius and EAS muscle thickness as described previously^32–34^.

Animals were subjected to EAS myotomy (as shown in the schematic, (Fig. 4B) as described previously^9^ and animals were allowed to recover. Prior to myotomy and immediately afterwards, the anal canal pressure recordings in response to EAS muscle electrical stimulation were obtained to study the length-tension (L-T) property of the EAS muscle, as described earlier^32–34^. Anal canal pressure/L-T measurements were repeated two times a month for 15 weeks.

### Pharmacological interventional studies with Wnt Antagonist

To test the hypothesis that Wnt inhibition will attenuate collagen deposition and improve EAS muscle function, a group of rabbits were subjected to EAS myotomy (experimental; n = 12), immediately after which they received a local injection at the myotomy site that consisted of either a Wnt antagonist, secreted frizzled receptor protein, sFRP-2 (R&D systems, MN), 2 µg in 200 µl saline (n = 6) or an equal volume of 0.9% saline (placebo), (n = 6). Injection was blinded to minimize operator bias and repeated every day for 7 days at the myotomy site. Animals were sacrificed at the end of the study (15 weeks) and the anal canal was harvested and fixed in formalin to perform the following tissue analysis: (1) Histology & histochemical evaluation to determine fibrosis/collagen content^35^. (2) Immuno-histochemistry/ immuno-fluorescence to localize proteins involved in fibrosis/connective tissue formation. (3) Western blots to quantify relevant proteins. (4) Quantitative PCR to determine changes in gene expression.

#### Histological Evaluation

Skin was removed and anal canal sections were processed. Paraffin tissue sections (7 µm) were applied to the microscopic slides, deparaffinized and stained with Masson- trichrome (muscle/connective tissue) and Sirius red stain (for collagen). Digital images of the entire anal canal cross section (muscle & mucosa) were captured and image analysis was performed to quantify connective tissue/collagen and skeletal muscle components (*Nikon NIS Elements*, *Melville*, *NY*)^36^. Muscle and connective tissue components were identified by red and blue color stain respectively. Entire anal canal sections were imaged (10X) and images were quantitated for the red pixels (stained for skeletal muscle) and blue pixels (connective tissue components) as percentage of the total number of pixels.

#### Immunostaining studies

We performed immunofluorescence (IF) studies to localize β-catenin and immunohistochemistry (IHC) to localize collagen-I. Paraffin tissue sections were processed for antigen retrieval. Sections were incubated for 30 min with 5% normal goat/horse serum containing 1% Triton X-100 in order to block the nonspecific binding sites. These sections were further incubated overnight at 4 °C with specific monoclonal antibodies for β-catenin (Abcam), (1:200) dissolved in the PBS containing 1% serum. In one set, tissues were incubated with normal mouse IgG in the absence of primary antibody, which served as a negative control. After three washings, sections were further incubated for 2 hours with appropriate anti-mouse secondary antibodies and conjugated with rhodamine. Incubation was terminated by washing with PBS and the slides were mounted in Gel/Mount. The slides were kept in dark at 4 °C and observed under a fluorescent microscope within 24 hours for imaging. For IHC studies, sections were subjected to antigen retrieval followed by incubation with the primary antibody (collagen-I; Abcam; 1:200) and processed using vectastain peroxidase system (Vector labs, CA) to localize collagen- 1.

#### Western Blot

To quantify levels of fibrogenic markers (β-catenin, Collagen-I and TGF-β1), equal amounts of protein (25 μg) were loaded onto a 12% polyacrylamide gel (29:1) along with a pre-stained molecular weight standard. After overnight transfer to nitrocellulose membrane in 25 mM Tris–192 mM glycine–20% methanol transfer buffer, the nonspecific binding sites were blocked with 5% milk in PBS, pH 7.5. The membrane was then incubated overnight with appropriate primary antibodies. After washing in PBS, the blots were incubated with an appropriate secondary antibody for 30 min followed by another washing and then incubated for 5 minutes in super signal chemiluminescent substrate. The signal were detected on X-O mat AR film and processed in an automatic film developer. Protein bands were quantified using an image analysis program (Scion Image, Scion Corporation, Frederick, MD, USA) and the intensities normalized to the amount of protein in the supernatant^26, 37^.

#### Quantitative PCR

Total RNA was extracted from control and EAS injured rabbit samples (n = 3–4 each) using RNA STAT-60, treated with RNase-free DNase to eliminate genomic DNA contamination, and purified with RNeasy Mini kit (Qiagen, Valencia, CA). The cDNA was synthesized from 4 μg total RNA by reverse transcription reaction using SuperScript II reverse transcriptase (Invitrogen, Carlsbad, CA) and random hexamers (Invitrogen). The primer pairs used for quantitative RT-PCR analysis of TGF-β1, β-catenin and collagen-I have been previously reported^38–40^ β-catenin -Forward: 5′-ATG TGG ATT TGG AAC CCA AG-3′; Reverse: 5′-CCA AAG GGA GGC TTC CTA GT-3′. Collagen I- Forward: 5′-TAG GCG TTC CAG TTC GAG TA-3′; Reverse: 5′GGT CTT CCG GTG GTC TTG TA-3′; TGF-β1-Forward: 5′-ACA TTG ACT TCC GCA AGG AC-3′; Reverse: 5′-TAG TAC ACG ATG GGC AGT GG-3′. GAPDH-Forward-5′-GCA CCG TCA AGG CTG AGA AC-3′; reverse: 5′-ATG GTG GTG AAG ACG CCA GT-3′. Specificity of each RT-PCR reaction was checked by its dissociation curve. Single product amplification and correct product size were confirmed by agarose gel electrophoresis. Quantitative real-time PCR was conducted on a Mx3000P QPCR System (Stratagene, La Jolla, CA) using iQ SYBR Green Supermix (Bio-Rad, Hercules, CA) under the following conditions: 5 min at 98 °C, 40 cycles of 30 s at 95 °C, 30 s at 55 °C, and 30 s at 72 °C. RNA equivalents were normalized to simultaneously determine glyceraldehyde-3-phosphate dehydrogenase (GAPDH) mRNA levels in each sample.

### Data analysis

All pressures were measured and analyzed as described previously. The force of contraction of the EAS muscle was determined by calculating muscle tension (Tm) using the following equation: Tm = P × rm/tm, where P is the intraluminal pressure, rm is the inner radius of the EAS muscle, and tm is the EAS muscle thickness. The rm and tm were measured from the ultrasound images of the rabbit anal canal and further confirmed by measuring these parameters in a freshly harvested specimen. EAS muscle thickness was calculated for each probe size assuming conservation of mass. The EAS muscle tension represents the average circumferential force per unit area of the circular muscle and is denoted in milli-newton per centimeter squared (mN/cm^2^)^31^.

### Statistical analysis

Data are shown as means ± SE. For length-tension studies, a one way repeated-measure ANOVA with post hoc Tukey’s test (SPSS) was performed. For all other comparisons muscle/connective tissue evaluations. one-way ANOVA followed by Bonferroni post-hoc test was employed.

## Electronic supplementary material

Supplementary Information
