# Supplementary figures and images for: Wnt-β Catenin Signaling Pathway: A Major Player in the Injury Induced Fibrosis and Dysfunction of the External Anal Sphincter
Source: Sci Rep. 2017 Apr 19;7:963. doi: 10.1038/s41598-017-01131-6 (PMC5430485; doi:10.1038/s41598-017-01131-6)

$\beta$ -Catenin

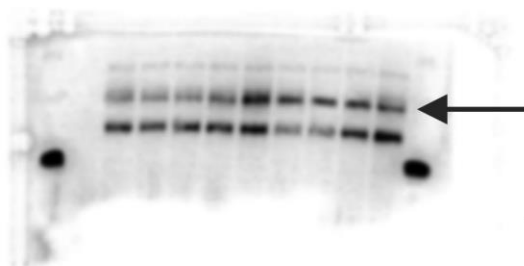

Collagen-1

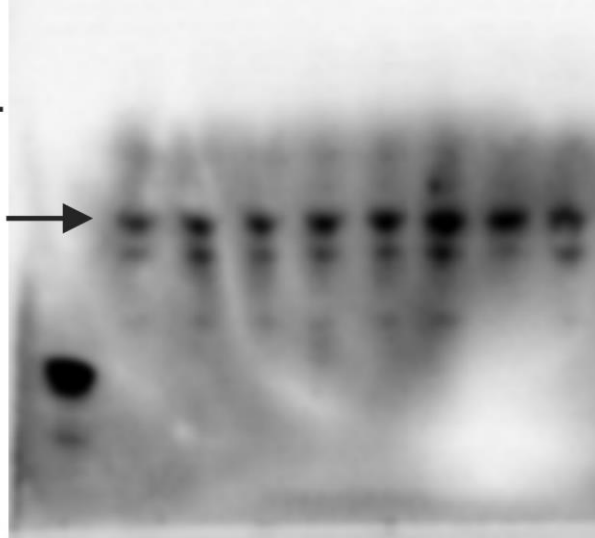

TGF $\beta$

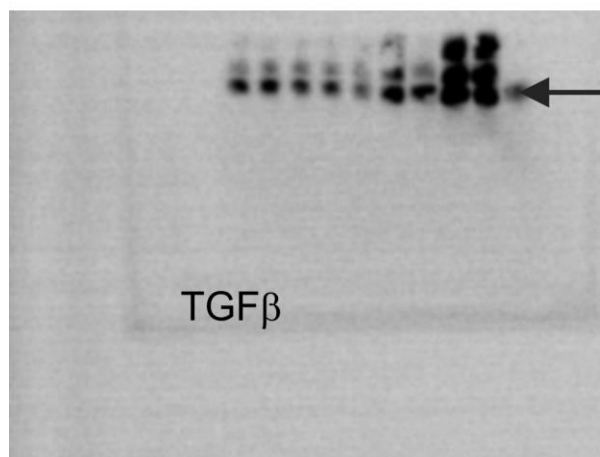

GAPDH

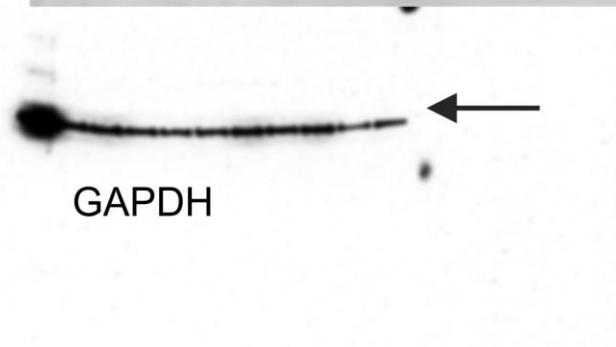

Supplement: Supplementary file 1 — Supplementary Information [file 41598_2017_1131_MOESM1_ESM.pdf]
